# A promising structure for fabricating high strength and high electrical conductivity copper alloys

**DOI:** 10.1038/srep20799

**Published:** 2016-02-09

**Authors:** Rengeng Li, Huijun Kang, Zongning Chen, Guohua Fan, Cunlei Zou, Wei Wang, Shaojian Zhang, Yiping Lu, Jinchuan Jie, Zhiqiang Cao, Tingju Li, Tongmin Wang

**Affiliations:** 1 Key Laboratory of Solidification Control and Digital Preparation Technology (Liaoning Province), School of Materials Science and Engineering, Dalian University of Technology, Dalian, 116024, China; 2 Laboratory of Special Processing of Raw Materials, School of Materials Science and Engineering, Dalian University of Technology, Dalian, 116024, China; 3 School of Materials Science and Engineering, Harbin Institute of Technology, Harbin, 150001, China

## Abstract

To address the trade-off between strength and electrical conductivity, we propose a strategy: introducing precipitated particles into a structure composed of deformation twins. A Cu-0.3%Zr alloy was designed to verify our strategy. Zirconium was dissolved into a copper matrix by solution treatment prior to cryorolling and precipitated in the form of Cu_5_Zr from copper matrix via a subsequent aging treatment. The microstructure evolutions of the processed samples were investigated by transmission electron microscopy and X-ray diffraction analysis, and the mechanical and physical behaviours were evaluated through tensile and electrical conductivity tests. The results demonstrated that superior tensile strength (602.04 MPa) and electrical conductivity (81.4% IACS) was achieved. This strategy provides a new route for balancing the strength and electrical conductivity of copper alloys, which can be developed for large-scale industrial application.

Today’s high-speed trains demand extremely well-designed copper materials that should simultaneously possess high strength and high electrical conductivity to manufacture the railway contact wire^1^. For copper and its alloys, however, high strength and high electrical conductivity are mutually exclusive in nature; therefore, an either-or situation must be taken into account between the properties in the alloy design^2^. Although the rapid development of high-speed technology in recent years has promoted the emergence of many specifications, improving the strength of copper-based materials without impairing the electrical conductivity remains challenging in practice. To date, precipitation, grain refinement and twinning strengthening are the most common processing methods that are used to keep a desired balance between the mechanical strength and the electrical conductivity. Precipitation strengthening is generally achieved via thermo-mechanical treatment^3^, the precipitates thus formed can act as obstacles to the movement of dislocations in the matrix, thereby significantly strengthening the copper matrix. However, the drawback is that after the thermo-mechanical treatment, if the solute elements are not fully separated out from the matrix, then they are prone to reduce the electrical conductivity of the copper matrix. In addition, the finite solute content limits the improvement of the strength. Grain refinement is often performed via various severe plastic deformation techniques, such as equal channel angular pressing^4^, accumulative rolling bonding^5^, and high pressure torsion^6^. The strengthening mechanism of grain refinement relies on incoherent grain boundaries to obstruct dislocation movement, as described by the Hall-Petch relationship. Unfortunately, an increasing number of grain boundaries severely exacerbate the scattering of conducting electrons, thereby impairing the electrical conductivity of the material^2^.

Unlike the precipitation and grain refinement strengthening, twinning strengthening appears to be the most promising route for producing high strength and high electrical conductivity copper alloys because twin boundaries can effectively block the dislocation motion while having a negligible effect on the electrical conductivity^2^,^7^. Twinning strengthening can be obtained via the pulsed electrodeposition technique (growth twin)^2^ and dynamic plastic deformation (deformation twin)^8^. Of the deformation twins, an advantageous aspect is that a twin fragment may directly result in grain refinement, which in turn leads to marked reduction in the grain size down to dozens of nanometres^9^. Grain size, stacking fault energy (SFE), deformation temperature and strain rate are considered the key factors that determine the formation of deformation twins^10^. Increasing the strain rate and decreasing the plastic deformation temperature and SFE of the matrix promote the formation of more deformation twins. In this regard, the bulk fabrication of nano-grained copper with a tensile yield strength of 610 MPa and an electrical conductivity of 95% IACS has been prepared via dynamic plastic deformation at cryogenic temperature^7^. Although the twin boundary has a significant strengthening effect^11^,^12^, the interiors of the twin/matrix lamellae and internals between twin bundles are “weak zones” because of the weak dislocation-dislocation interaction. Only when the twin/matrix lamellar thickness falls in the nanometre scale does the twin boundary contribute significantly to the strength improvement. This requirement limits the application of twinning strengthening in industrial structural alloys^13^. In addition, most nano-twinned copper alloys induced by plastic deformation are solid-solution strengthened copper alloys with low SFE, such as Cu-Zn alloys^14^, Cu-Al alloys^15^, and Cu-Al-Zn alloys^16^. Although these alloys have superior mechanical properties, their shortcoming is that the solutes inevitably deteriorate the electrical conductivity.

To solve the above-described problems, we propose a strategy: introducing precipitated particles into a structure composed of deformation twins. Cryorolling, i.e., rolling at liquid nitrogen temperature, was utilized to “freeze” the movement of the dislocations, and restrict dislocation cross-slip and climb to planar dislocation glide, These effects promote the formation of deformation twins. Compared with dynamic plastic deformation, cryorolling has a great potential for continuous commercial application^17^,^18^,^19^. Under this scheme, the alloy element should have a low solubility in copper matrix at room temperature and the potential to lower the SFE of the copper matrix. Once it is dissolved in the copper matrix, the alloy element should not be separated out and should act as an agent to produce more deformation twins during the cryorolling, after which the added element should precipitate in the form of intermetallic compounds from the copper matrix via subsequent aging treatment. The precipitates hinder the movement of twin boundaries during aging treatment and improve the strength of matrix inside the lamellae after aging treatment. More importantly, the diminished solute content has limited adverse effects on the electrical conductivity of the final material.

Zirconium (Zr) was chosen to verify this strategy. It is suggested that the addition of Zr will lower the SFE of copper matrix^20^, thereby promoting the formation of deformation twins. In addition, the solubility of Zr in copper matrix at room temperature is only 0.01% (all in mass fraction unless otherwise specified), such a low level that has minor influence on the electrical conductivity of the copper matrix. Our strategy provides a new route to develop copper alloys with the integrated strength and electrical conductivity. The method is deemed to have great potential for large-scale industrial application. This approach may also be appropriate for other precipitation strengthened copper alloys, such as Cu-Cr alloys, Cu-Cr-Zr alloys, and Cu-Ni-Si alloys.

## Methods

The nominal composition Cu-0.3% Zr alloys were prepared by mixing electrolytic copper (99.99%) and Cu-49.9% Zr alloy in a vacuum induction furnace under argon atmosphere. Casting was performed using a metal mould with a size of 40 × 50 × 150 mm^3^. After homogenising at 900 °C for 5 hours and removing the oxidation layer, the samples were hot rolled from 30 mm to 20 mm with 33.33% thickness reduction at 850 °C. The hot rolled samples were solution treated at 972 °C for 1 hour prior to water quenching. After removing the oxidation layer and the surface defects, the sample was cryorolled from 18 mm to 1.5 mm with 91.7% thickness reduction. A reference sample, rolled at room temperature (20 °C), was also prepared for comparison. Cryorolling was performed by immersing the samples into liquid nitrogen till they have reached the saturation temperature (−196 °C). Prior to cryorolling, each sample was dipped in liquid nitrogen for 10 min. Multiple passes were applied to deform the samples with 10% reduction for each pass, after which the plates were dipped in liquid nitrogen immediately and immersed for 5 min before further reduction. The isochronal aging at various temperatures for 1 hour and isothermal aging at 350 °C, 400 °C and 450 °C for various times were performed in a tubular electric resistance furnace with a sample size of 20 × 20 mm^2^. The microstructures were characterised using X-ray diffraction (XRD) and transmission electron microscopy (TEM). The XRD measurements were performed using an EMPYREAN diffractometer equipped with a Cu radiation target. Pure copper powder (99.95%) annealed at 400 °C was used as an XRD peak-broadening reference for the microstrain calculations. Discs of 3 mm in diameter punched from a longitudinal section (Nominal direction-rolling direction plane) of the plates were ground to 30 μm and were then double jet thinned using a 25% nitric acid in methanol solution at −30 °C. TEM observations were performed using a transmission electron microscope operating at 300 kV (Tecnai G2 F30). Vickers micro-hardness (HV) measurement was conducted by applying a load of 300 g for 15 s on the rolling plane using a MH-50 type micro-hardness tester. Samples with a parallel length of 40 mm were tensile-tested on an Instron 5500R universal tensile machine with an initial strain rate of 2 mm/min at 298 K. The electrical conductivity was measured using a D60K digital electrical instrument.

## Results

### Microstructures

**Figure 1:**
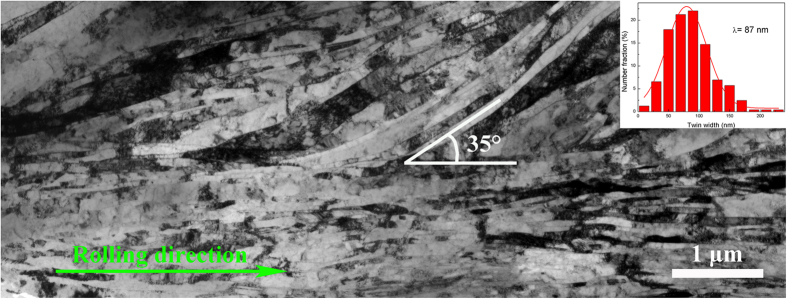
A cross-sectional bright field TEM image of RTR Cu-Zr alloys. The inset shows the statistics distribution of the twin/matrix lamellar thickness in RTR sample.

**Figure 2:**
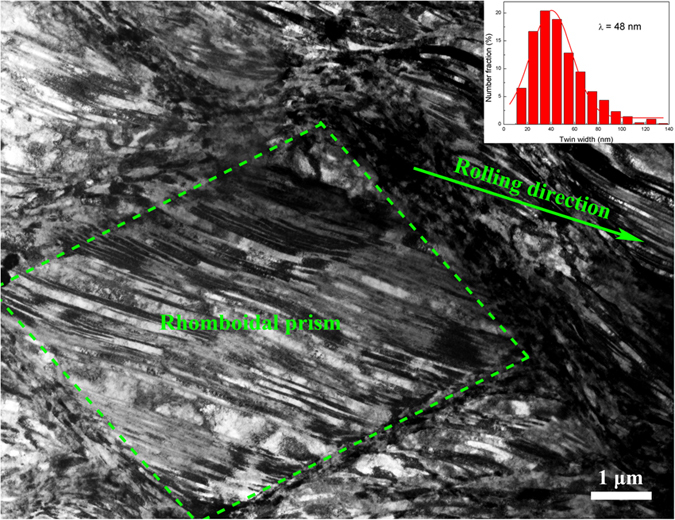
A cross-sectional bright field TEM image of CR Cu-Zr alloys. The inset shows the statistics distribution of the twin/matrix lamellar thickness in CR sample.

**Figure 3:**
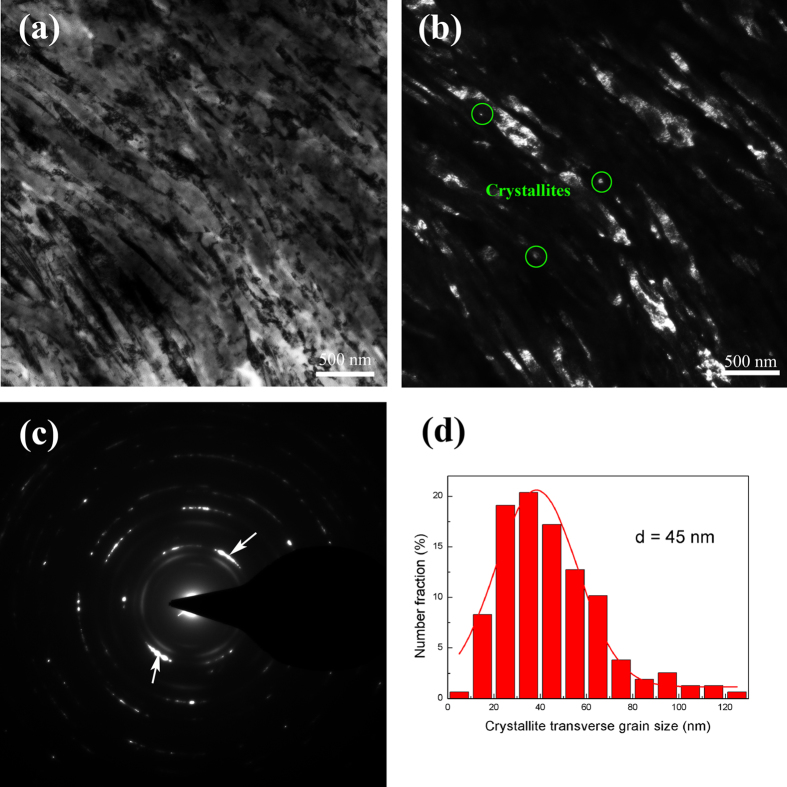
TEM images of nano-size crystallites evolved from twin/matrix lamellae of CR Cu alloys. (**a**) A bright field image; (**b**) a dark field image; (**c**) an SAED pattern corresponding to (**a**,**d**) statistic distribution of transverse grain size.

Figure 1 shows typical cross-sectional bright field TEM images of room temperature rolled (RTR) Cu-Zr samples. When they were rolled at room temperature, the solution-treated Cu-Zr alloys were pancaked into flat grains. Thus, elongated grains parallel to rolling direction can be acquired; the average transverse grain size is approximately 216 nm. A small volume fraction of deformation twins also exists in the RTR samples. The inset in Fig. 1 shows the thickness distributions of the twin/matrix lamellae. The average twin/matrix lamellar thickness of the RTR samples is 87 nm. The shear bands inclined to rolling direction approximately 35° “cut” the matrix. Elongated lamellae, deformation twins and shear bands are representative microstructures in RTR Cu-Zr alloys. The cross-sectional TEM image of cryorolled (CR) sample is shown in Fig. 2. Many deformation twins in the form of bundles are detected in CR samples, whereas elongated lamellae parallel to rolling direction are rarely observed. Most of the twin boundaries are curved and contain a large number of dislocations, which are the characteristics of deformation twins. Statistical measurements show the average twin/matrix lamellar thickness of CR samples (the inset in Fig. 2) is 48 nm, which decreases nearly 45% compared with that of the RTR sample. Another significant feature of CR samples is the development of a rhomboidal prism that has an axis parallel to the transverse direction, which is defined by two sets of shear bands. In this case, the shear bands “cut” through the deformation twin regime. Figure 3a shows the twin/matrix lamellar microstructures in the vicinity of shear bands. The nano-twin bundles are curved and bent toward the shear direction. “Bamboo-like” nano-size crystallites that evolved from twin/matrix lamellae of CR Cu-Zr alloys are observed in the dark field image (Fig. 3b). Most of the crystallites are elongated and some are equiaxed. From the selected area electron diffraction (SAED) pattern shown in Fig. 3c, the misorientations of crystallites are small and the strong maxima on the ring of the {111} reflection clearly indicates a preferential orientation of the grains (as shown in the white arrows). The statistical distribution of the transverse grain size is shown in Fig. 3d. The mean transverse grain size is 45 nm, which is close to the thickness of the twin/matrix lamellae.

**Figure 4:**
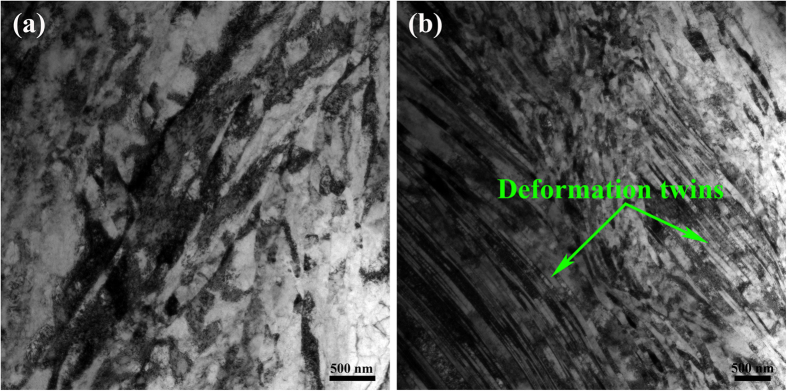
TEM images of RTR (**a**) and CR (**b**) Cu-Zr alloys aged at 350 °C for 120 min.

**Figure 5:**
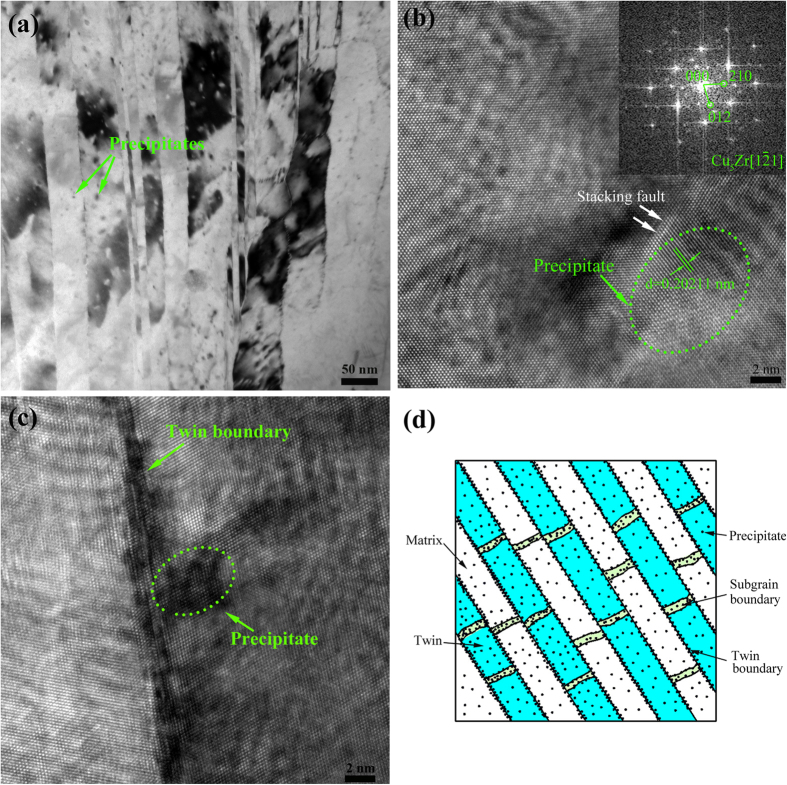
(**a**) A bright field TEM image of CR Cu-Zr alloys aged at 400 °C for 120 min; (**b**) a HRTEM image of the precipitates in the matrix; the inset shows the FFT image along a [1–21] zone axis of Cu_5_Zr; (**c**) a HRTEM image of the precipitates at the twin boundary; (**d**) schematic diagram of the strengthening model combining deformation twins and precipitates.

The TEM bright images of RTR and CR Cu-Zr alloys aged for 120 min at 350 °C are shown in Fig. 4. Although recovery occurs and the densities of dislocations decrease in both rolling conditions, large numbers of deformation twins still remain in the CR samples. Figure 5a describes a bright field TEM image of CR Cu-Zr alloys aged at 400 °C for 120 min. It can be observed that nano-scale precipitates are distributed within the twin/matrix lamellae. Figure 5b shows a typical coherent precipitate in the copper matrix (marked by a green dotted line ellipse), which is confirmed by the fast Fourier transformation (FFT) image (the inset in Fig. 5b). The interplanar spacing of the precipitate is 0.20211 nm, which is consistent with the (222) plane of Cu_5_Zr. The stacking faults induced by stress were observed neighbouring the interface between the precipitate and matrix (marked by white arrows in Fig. 5b). Fine precipitates were also observed at the twin boundary (Fig. 5c). The combination structures of deformation twins and precipitates are schematically depicted in Fig. 5d.

### Mechanical and electrical conductivity properties

**Figure 6:**
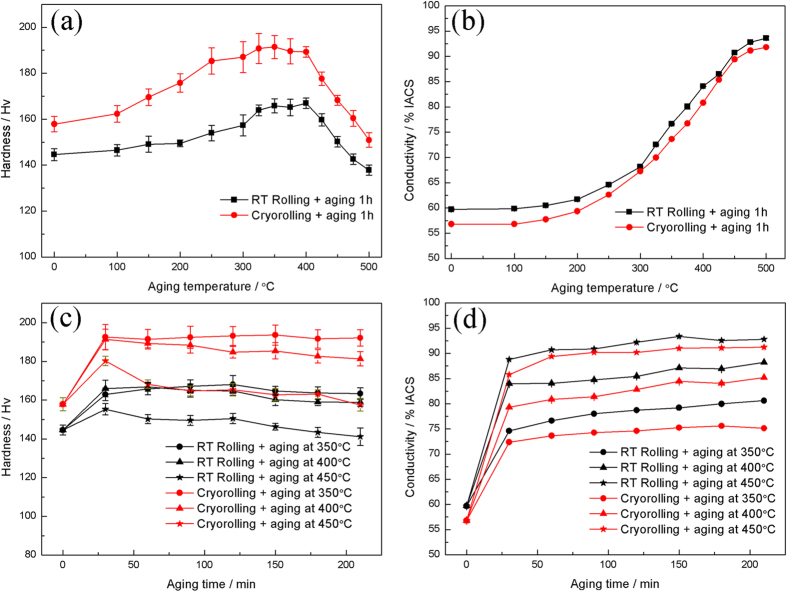
Variation of hardness and electrical conductivity of CR and RTR Cu-Zr alloys during 1 h isochronal and isothermal aging. (**a**) Variation of hardness during 1 h isochronal aging; (**b**) variation of electrical conductivity during 1 h isochronal aging; (**c**) variation of hardness during isothermal aging; (**d**) variation of electrical conductivity during isothermal aging.

**Figure 7:**
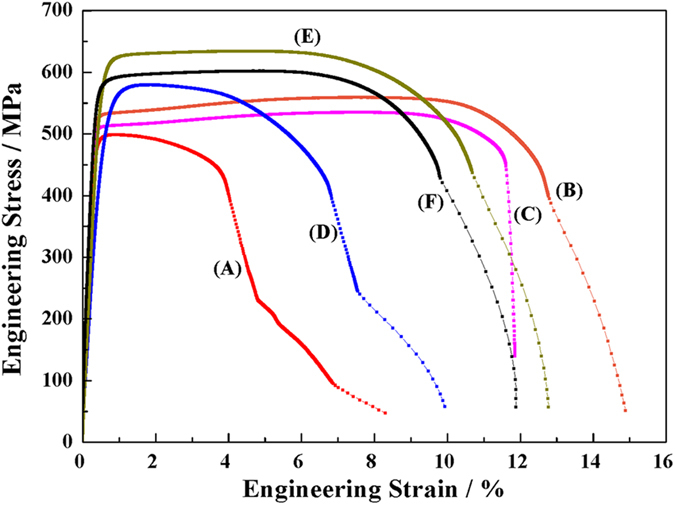
Engineering Stress-strain curves of Cu-Zr alloys subjected to different aging conditions. (**A**) RTR sample; (**B**) RTR sample, aging at 350 °C for 120 min; (**C**) RTR sample, aging at 400 °C for 90 min; (**D**) CR sample; (**E**) CR sample, aging at 350 °C for 120 min; (**F**) CR sample, aging at 400 °C for 90 min.

To obtain superior integrated mechanical properties and electrical conductivity, isochronal and isothermal aging treatments were conducted. The variations of the hardness and the electrical conductivity of CR and RTR Cu-Zr alloys against isochronal and isothermal aging are shown in Fig. 6. The maximum hardness is obtained at 350 °C after aging for 120 min in both conditions. Considering both the hardness and the electrical conductivity, the suitable condition was fixed with aging at 400 °C for 90 min. Figure 7 shows the engineering stress-strain curves of the CR and RTR Cu-Zr alloys that were subjected to different aging conditions. The detailed mechanical and electrical conductivity properties of RTR and CR Cu-Zr alloys are listed in Table 1. After a 91.5% rolling reduction, the ultimate tensile strength of the CR samples (535.97 MPa) is much larger than that of the RTR samples (498.60 MPa). The uniform elongation of CR samples is also improved compared with that of the RTR samples. After aging treatment, the tensile strength saw an increase with respect to the as-rolled samples. The highest tensile strength of the CR Cu-Zr alloys is 634.49 Mpa, whereas that of the RTR sample is merely 559.35 MPa. The desired integration of tensile strength (602.04 MPa) and electrical conductivity (81.4% IACS) is achieved by cryorolling and subsequent aging at 400 °C for 90 min. Compared with traditional room temperature rolling, the tensile strength of the CR samples was increased by 12.5%, and electrical conductivity was decreased by only 3.3% IACS.

## Discussion

Slipping and twinning are two competing processes in plastic deformation. Grain size, SFE, temperature and strain rate are considered the four key factors that determine the tendency to form deformation twins. Twin stress mainly depends on grain size and follows a Hall-Petch relationship^21^. With an initial as-cast grain size of 134.9 μm, Cu-Zr alloys have a great potential for twinning. In addition, the strain rate of rolling is not as high as dynamic plastic deformation. As a result, the effects of grain size and strain rate on deformation twins are neglected in our case. In this study, we focus on the effect of SFE and rolling temperature on the microstructure evolution during rolling. As we know, the formation of deformation twin consists of two processes: the nucleation and growth of twin embryos. The critical twin nucleus thickness, *λc*, can be described by the following equation^22^:


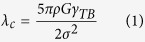


where *G* is the shear modulus, *ρ* is the ratio of thickness to diameter of the twin embryo (a constant), *γ*_*TB*_ is the twin boundary energy (which is proportional to the SFE), and *σ* is the driving stress of twin nucleation. Due to the different crystal structure of Zr (hexagonal close packed) and Cu (face-centred cubic), the addition of Zr in small quantities significantly promotes the decrease in the SFE of copper matrix^23^. The lower SFE of Cu-Zr alloys leads to a smaller critical twin nucleus thickness (*λc*). Thus, it is easier to form deformation twins in RTR Cu-Zr alloys compared with pure copper. The driving stress of twin nucleation (*σ*) increases with the increase in the strain rate and/or the decrease in temperature for face-centred cubic alloys^22^. It is obvious that CR Cu-Zr alloys have a larger volume fraction of deformation twins than RTR alloys. Due to the lower twin boundary energy and higher driving stress of Cu-Zr alloys that are subjected to cryorolling, twinning dominates the deformation process. Decreasing rolling temperature not only increases the volume fraction of deformation twins but also decreases the thickness of the twin/matrix lamellae. The mean twin/matrix lamellar thickness of the CR samples is 48 nm, whereas that of RTR samples is 87 nm. Dislocation movement is a thermally activated process. During cryorolling, the dislocation recovery is effectively suppressed. Dislocations cannot move by cross slip or climb. Regarding high SFE materials, cryorolling enhances the efficiency of plastic deformation, and a high density of dislocations is restored in the matrix. The high defect density leads to the increase of nucleation sites, which promotes the development of ultrafine grained materials during annealing^24^. Regarding the materials with medium to low SFE, the movement of dislocations is restricted to planar glide during cryorolling, which promotes the accumulation of stress for twinning. Lowering both the SFE and the deformation temperature increases the density of deformation twins. Therefore, the addition of Zr and the decreased rolling temperature play key roles in determining the formation of deformation twins.

Shear banding is a consequence of localized deformation. When work hardening cannot be achieved by homogenous plastic deformation, shear banding will dominate the deformation mode. Many factors such as the strain rate, deformation temperature and SFE determine the features of shear bands^25^. There are two types of shear bands: copper and brass. The former is based on a dislocation matrix and “cuts” the microbands of the matrix (as shown in Fig. 1), which usually exists in heavy deformed metals with a high or medium SFE, especially in heavy deformed copper. Although the addition of Zr lowers the SFE and promotes the formation of deformation twins, the SFE of Cu-Zr alloys is not sufficiently low to change shear band type. The latter is based on twinning matrix and “cuts” deformation twin bundles (as shown in Fig. 2), which usually exists in metals with medium to low SFE. As indicated in Fig. 2, the deformation twins were divided into prisms with axes parallel to the transverse direction. This prism feature verifies the significance of Zr and rolling temperature.

The formation mechanism of nano-size crystallites is the fragmentation of twin/matrix lamellae. Manipulation and rearrangement of dislocations dominate the further deformation to accommodate plastic strain within the nano-size bundles because it is difficult for deformation twins to nucleate^26^. The accumulated dislocations within the twin/matrix lamellae and at the twin boundaries subdivide the continuous twin bundles into many discontinuous cell blocks. With the increase of strain, these refined cell blocks evolve to sub-grains with low and/or high misorientations. Differing from the random crystallites in dynamic plastic deformed copper^8^, the crystallites in CR Cu-Zr alloys perform preferential orientation, and the misorientations are rather small. This behaviour is ascribed to the fact that the stress in the nano-twin bundles is not sufficient to form randomly distributed nano-scale crystallites. Further straining or a reduction of the rolling temperature will produce more refined and random distributed nano-scale crystallites. Although some deformation twins are fragmented to discontinuous nano-size crystallites, others retain a continuous structure.

Although precipitates are not observed after aging at 350 °C for 120 min, the precipitation process does occur. The precipitates prefer to nucleate in the severely deformed zones with a large number of defects. The low aging temperature also limits the growth of precipitates. These two factors are responsible for the lack of observed precipitates. In addition, the resistivity of copper alloys increases linearly with the solute content^3^. The increase of electrical conductivity of the aged samples verifies the existence of precipitates. However, large numbers of nano-scale disk-like precipitates are visible in the CR Cu-Zr alloys aged at 400 °C for 90 min (Fig. S1 in the Supplementary Information online). The precipitates are identified as Cu_5_Zr according to interplanar spacing and the FFT image in Fig. 5b, which is consisted with Peng *et al.*^27^ and Wang *et al.*^28^. As depicted in Fig. 5d, the precipitates are distributed at twin boundaries, sub-grain boundaries, and within the twin/matrix lamellae. The twin boundaries are more stable than dislocations and grain boundaries in nature, and the precipitates at twin boundaries (Fig. 5c) can pin the movement of twin boundaries during aging treatment. Therefore, deformation twins remained stable after aging treatment as the dislocations began to annihilate (Fig. 4). The traditional twinning strengthening is based on twin boundaries and grain refinement by twin fragments. The dislocation activities within the twin/matrix lamellae and internals between twin bundles are blocked by dislocation-dislocation interaction. Therefore, the interiors of twin/matrix lamellae and internals between twin bundles are “weak zones” compared with the twin boundaries. Our structure model makes up these “weak zones” and replaces the dislocation-dislocation interaction with dislocation-precipitate interaction. The precipitates not only maintain the high temperature stability of deformation twins but also strengthen the “weak zones”. More importantly, the precipitates reduce the solute content in copper matrix. Thus, the strength and electrical conductivity are simultaneously improved. Generally, this structure of the combination of deformation twins and precipitates is suitable for fabricating high strength and high electrical conductivity copper alloys in practice.

As noted above, cryorolling decreases the twin/matrix lamellar thickness and increases the volume fraction of deformation twins. These profuse nano-scale twins can effectively improve the strength. In addition, the fine crystallites derived from fragments of deformation twins play a role in improving the strength. Therefore, twinning and grain refinement strengthening are responsible for the improved strength of CR samples compared with RTR ones. After aging treatment, the dislocation-twin boundary and dislocation-precipitate interactions within twin/matrix lamellae and internals between twin bundles contribute to the increased strength of the CR samples compared with the RTR samples. The cryorolling process improves not only the strength but also the uniform elongation of Cu-Zr alloys. Yu *et al.* attributed this phenomenon to the precipitation from Al matrix during room temperature rolling, which simultaneously decreased the solute content and increased the SFE^29^. However, precipitations are not observed in either the RTR or CR samples in the TEM images. In addition, the low solid solubility of Zr in the copper matrix plays a minor role in the improving ductility^9^. The inability to accumulate dislocations limits the work hardening. The dislocation densities (*ρ*_*d*_) can be calculated using the following equation^30^.


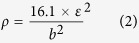


Where *ε* is the microstrain, and *b* is the Burgers vector. The dislocation densities of various conditions are listed in Table 1. The increases in uniform elongation are always accompanied by decreases in dislocation densities, which is in accordance with previous reports^31^. Huang *et al.* argued that when deformation twinning became activated, the uniform elongation began to increase^9^. As shown in Figs 1 and 2, cryorolling changes the plastic deformation mode and large numbers of deformation twins are developed in the CR samples. The lower dislocation density of the CR samples also indicates that intensive twinning bears the majority of the plastic deformation and that the contribution of dislocation slip is reduced during cryorolling^32^. Therefore, the deformation twins account for the improved ductility of CR Cu-Zr alloys compared with the RTR ones. When suffering from aging, the RTR and CR samples experience recovery, with dislocations annihilating and rearranging themselves into a configuration of low energy. This process facilitates the further accumulation of dislocations and leads to an improvement in ductility. During this process, the dislocation density of the RTR samples decreases rapidly compared with that of the CR samples (see Fig. 7 and Table 1). This result is attributed to the interaction between dislocations and twin boundaries, which inhibits the dislocation movement.

According to Mattiessen’s rule, the total resistivity of a metal (*ρ*_*total*_) can be expressed by


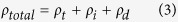


Where *ρ*_*t*_, *ρ*_*i*_, and *ρ*_*d*_ are the contributions from thermal vibrations, impurities, and lattice defects, respectively^2^. The solute Zr atoms in the copper matrix act as impurity centres for the scattering of electron motions and thus significantly deteriorate the electrical conductivity. As a result, the electrical conductivity of solution treated sample is only 55.7% IACS. Although lattice defects such as dislocations and grain boundaries hinder the movement of conduction electrons, the electrical conductivity increases slightly after room temperature rolling and cryorolling. This phenomenon may be attributed to the fact that a very small quantity of the solute atoms precipitates from the supersaturated solid solution during deformation. A further analysis indicates that the electrical conductivity values of the CR samples subjected to different aging conditions are slightly lower by approximately 3–5% IACS compared with those of the RTR samples that underwent the same aging treatment. The great difference between the CR and RTR samples with the same aging conditions is the number of lattice defects. Comparing the electrical conductivity and the dislocation density of the CR and RTR Cu-Zr alloys in Table 1, the effect of dislocation density on the electrical conductivity can be negligible. The slight decrease of the electrical conductivity of CR Cu-Zr may be ascribed to the increment of grain boundaries and twin boundaries, which is consistent with the results of ref. 2. According to Zhang *et al.*^7^, the amounts and characteristics of the grain boundaries, not the twin boundaries, determine the electrical conductivity. Therefore, the increase in the number of grain boundaries due to grain refinement during cryorolling results in the slight decrease in electrical conductivity.

## Conclusions

In summary, the addition of Zr and cryorolling contributed to the profusion of nano-scale deformation twins and the change of shear band type. After aging treatment, the precipitated particles were introduced into a structure composed of nano-scale deformation twins. The precipitates stabilized the twin boundaries during aging treatment and strengthened the “weak zone”. Based on this structure, the desired balance between tensile strength (602.04 MPa) and electrical conductivity (81.4% IACS) was achieved for a Cu-0.3% Zr alloy (mass fraction). Compared with the traditional thermo-mechanical treatment, the tensile strength was increased by 12.5%, whereas the electrical conductivity saw an only 3.3% IACS decrease. The slight decrease of electrical conductivity is attributed to the grain boundaries induced by grain refinement.

## Additional Information

**How to cite this article**: Li, R. *et al.* A promising structure for fabricating high strength and high electrical conductivity copper alloys. *Sci. Rep.* **6**, 20799; doi: 10.1038/srep20799 (2016).

## Supplementary Material

Supplementary Information

## Figures and Tables

**Table 1:** Mechanical and electrical conductivity properties of RTR and CR Cu-Zr alloys.

**Alloy**	**Aging condition**	***σ***_**0.2**_**(MPa)**	***σ***_***UTS***_**(MPa)**	_***εu***_**(%)**	_***ρd***_**(10**^**14**^**m**^**−2**^)	**Cond (%IACS)**
RTR Cu-Zr	unaged	493.04	498.60	0.91	11.8	59.70
	350 °C 120 min	530.72	559.35	7.79	3.66	78.73
	400 °C 90 min	510.84	535.11	7.71	3.9	84.73
CR Cu-Zr	unaged	535.97	579.72	1.82	6.14	56.80
	350 °C 120 min	606.60	634.49	4.71	5.25	74.60
	400 °C 90 min	579.64	602.04	4.85	4.42	81.40

## References

[b1] Wang Z. *et al.* Influence of dc electric current on the hardness of thermally aged Cu–Cr–Zr alloy. J. Alloy. Compd. 479, 303–306 (2009).

[b2] Lu L., Shen Y., Chen X., Qian L. & Lu K. Ultrahigh strength and high electrical conductivity in copper. Science 304, 422–426 (2004). 15031435 10.1126/science.1092905

[b3] Xia C. *et al.* Study of deformation and aging behaviors of a hot rolled–quenched Cu–Cr–Zr–Mg–Si alloy during thermomechanical treatments. Mater. Des. 39, 404–409 (2012).

[b4] Habibi A., Ketabchi M. & Eskandarzadeh M. Nano-grained pure copper with high-strength and high-conductivity produced by equal channel angular rolling process. J. Mater. Process. Tech. 211, 1085–1090 (2011).

[b5] Takata N., Lee S. H. & Tsuji N. Ultrafine grained copper alloy sheets having both high strength and high electric conductivity. Mater. Lett. 63, 1757–1760 (2009).

[b6] Dobatkin S. V., Gubicza J., Shangina D. V., Bochvar N. R. & Tabachkova N. Y. High strength and good electrical conductivity in Cu–Cr alloys processed by severe plastic deformation. Mater. Lett. 153, 5–9 (2015).

[b7] Zhang Y., Li Y. S., Tao N. R. & Lu K. High strength and high electrical conductivity in bulk nanograined Cu embedded with nanoscale twins. Appl. Phys. Lett. 91, 211901 (2007).

[b8] Li Y. S., Tao N. R. & Lu K. Microstructural evolution and nanostructure formation in copper during dynamic plastic deformation at cryogenic temperatures. Acta Mater. 56, 230–241 (2008).

[b9] Huang C. X. *et al.* The effect of stacking fault energy on equilibrium grain size and tensile properties of nanostructured copper and copper–aluminum alloys processed by equal channel angular pressing. Mater. Sci. Eng. A 556, 638–647 (2012).

[b10] Zhang Y., Tao N. R. & Lu K. Effects of stacking fault energy, strain rate and temperature on microstructure and strength of nanostructured Cu–Al alloys subjected to plastic deformation. Acta Mater. 59, 6048–6058 (2011).

[b11] Huang Q. *et al.* Nanotwinned diamond with unprecedented hardness and stability. Nature 510, 250–253 (2014). 24919919 10.1038/nature13381

[b12] Tian Y. *et al.* Ultrahard nanotwinned cubic boron nitride. Nature 493, 385–388 (2013). 23325219 10.1038/nature11728

[b13] Xiao G. H., Tao N. R. & Lu K. Microstructures and mechanical properties of a Cu–Zn alloy subjected to cryogenic dynamic plastic deformation. Mater. Sci. Eng. A 513–514, 13–21 (2009).

[b14] Kumar R., Dasharath S. M., Kang P. C., Koch C. C. & Mula S. Enhancement of mechanical properties of low stacking fault energy brass processed by cryorolling followed by short-annealing. Mater. Des. 67, 637–643 (2015).

[b15] Subramanya Sarma V., Sivaprasad K., Sturm D. & Heilmaier M. Microstructure and mechanical properties of ultra fine grained Cu–Zn and Cu–Al alloys produced by cryorolling and annealing. Mater. Sci. Eng. A 489, 253–258 (2008).

[b16] Bahmanpour H. *et al.* Effect of stacking fault energy on deformation behavior of cryo-rolled copper and copper alloys. Mater. Sci. Eng. A 529, 230–236 (2011).

[b17] Panigrahi S. K., Jayaganthan R. & Pancholi V. Effect of plastic deformation conditions on microstructural characteristics and mechanical properties of Al 6063 alloy. Mater. Des. 30, 1894–1901 (2009).

[b18] Yu H. *et al.* Asymmetric cryorolling for fabrication of nanostructural aluminum sheets. Sci. Rep. 2, 772 (2012). 23101028 10.1038/srep00772 PMC3480655

[b19] Yu H. *et al.* Fabrication of ultra-thin nanostructured bimetallic foils by Accumulative Roll Bonding and Asymmetric Rolling. Sci. Rep. 3, 2373 (2013). 23918002 10.1038/srep02373 PMC3734478

[b20] Pang Y. *et al.* Effects of Zr and (Ni, Si) additions on properties and microstructure of Cu–Cr alloy. J. Alloy. Compd. 582, 786–792 (2014).

[b21] Meyers M. A., Vohringer O. & Lubarda V. A. The onset of twinning in metals: a constitutive description. Acta Mater. 49, 4025–4039 (2001).

[b22] Zhang Y., Tao N. R. & Lu K. Effect of stacking-fault energy on deformation twin thickness in Cu–Al alloys. Scripta Mater. 60, 211–213 (2009).

[b23] Kapoor K., Lahiri D., Batra I. S., Rao S. V. R. & Sanyal T. X-ray diffraction line profile analysis for defect study in Cu-1 wt.% Cr-0.1 wt.% Zr alloy. Mater. Charact. 54, 131–140 (2005).

[b24] Shanmugasundaram T., Murty B. S. & Subramanya Sarma V. Development of ultrafine grained high strength Al–Cu alloy by cryorolling. Scripta Mater. 54, 2013–2017 (2006).

[b25] Hatherly M. & Malin A. S. Shear bands in deformed metals Scripta Metal. 18, 449–454 (1984).

[b26] Tao N. R. & Lu K. Nanoscale structural refinement via deformation twinning in face-centered cubic metals. Scripta Mater. 60, 1039–1043 (2009).

[b27] Peng L. *et al.* The phase transformation and its effects on properties of a Cu−0.12wt% Zr alloy. Mater. Sci. Eng. A 633, 28–34 (2015).

[b28] Wang T. *et al.* Effects of trace La additions on the microstructures and properties of nanoprecipitates strengthened Cu–Zr alloys. J. Mater. Res. 30, 248–256 (2014).

[b29] Yu H. *et al.* A new insight into ductile fracture of ultrafine-grained Al-Mg alloys. Sci. Rep. 5, 9568 (2015). 25851228 10.1038/srep09568 PMC4389191

[b30] Zhang Y., Tao N. R. & Lu K. Mechanical properties and rolling behaviors of nano-grained copper with embedded nano-twin bundles. Acta Mater. 56, 2429–2440 (2008).

[b31] Zhao Y. H., Zhu Y. T., Liao X. Z., Horita Z. & Langdon T. G. Tailoring stacking fault energy for high ductility and high strength in ultrafine grained Cu and its alloy. Appl. Phys. Lett. 89, 121906 (2006).

[b32] Stepanov N. *et al.* Effect of cryo-deformation on structure and properties of CoCrFeNiMn high-entropy alloy. Intermetallics 59, 8–17 (2015).

