# Supplementary material for: A promising structure for fabricating high strength and high electrical conductivity copper alloys
Source: Sci Rep. 2016 Feb 9;6:20799. doi: 10.1038/srep20799 (PMC4746762; doi:10.1038/srep20799)
Supplement: Supplementary Information [file srep20799-s1.doc]

**Supplementary Information**

**A promising structure for fabricating high strength and high electrical conductivity copper alloys**

**Rengeng Li**^1ⱡ^**, Huijun Kang**^2ⱡ^**, Zongning Chen**^2^**, Guohua Fan^3^, Cunlei Zou**^1^**, Wei Wang**^1^**, Shaojian Zhang**^1^**, Yiping Lu**^1^**, Jinchuan Jie**^2^**, Zhiqiang Cao**^1^**, Tingju Li**^2^**,**

**Tongmin Wang**^1^*****

^1^ Key Laboratory of Solidification Control and Digital Preparation Technology (Liaoning Province), School of Materials Science and Engineering, Dalian University of Technology, Dalian 116024, China

^2^ Laboratory of Special Processing of Raw Materials, School of Materials Science and Engineering, Dalian University of Technology, Dalian, 116024, China.

^3^School of Materials Science and Engineering, Harbin Institute of Technology, Harbin 150001, China

**Rengeng Li** and **Huijun Kang** contributed equally to this work.

**Corresponding email**: [tmwang@dlut.edu.cn](mailto:tmwang@dlut.edu.cn) (Tongmin Wang)


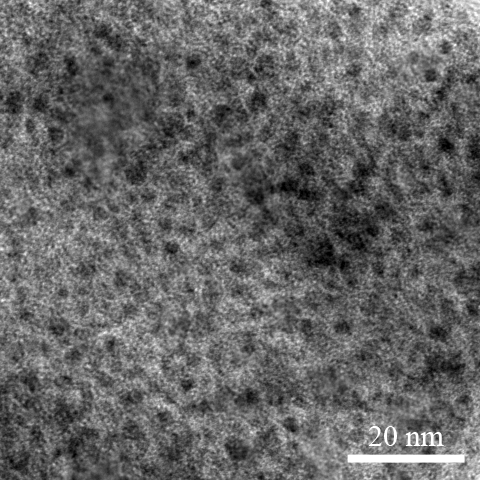


Fig. S1. TEM image of CR Cu-Zr alloys aged for 90 min at 400 ºC.
